# The Role of Leukemia Inhibitory Factor in Attenuating Skeletal Muscle Atrophy: Mechanisms to Exercise Interventions

**DOI:** 10.3390/cells15110981

**Published:** 2026-05-26

**Authors:** Na Jiang, Shiyi Wang, Jiaqiao Zhang, Dandan Jia

**Affiliations:** 1 Department of Physical Education, Suzhou University of Technology, Changshu 215500, China; 2 School of Exercise and Health, Shanghai University of Sport, Shanghai 200438, China

**Keywords:** leukemia inhibitory factor, exercise, skeletal muscle atrophy, hypertrophy, proliferation

## Abstract

**Highlights:**

**What are the main findings?**

- Exercise upregulates Leukemia inhibitory factor (LIF) in skeletal muscle, activating JAK2-STAT3, PI3K-Akt-mTOR, and MEK-ERK1/2 pathways to promote satellite cell activation, inhibit myocyte apoptosis, and drive physiological hypertrophy while suppressing muscle atrophy.
- LIF exerts diametrically opposed effects on skeletal muscle: myogenic LIF facilitates repair and growth, while tumor-derived LIF drives cancer cachexia-related muscle wasting…

**What are the implications of the main findings?**

- The LIF/LIFR axis serves as a potential therapeutic target for sarcopenia, muscular dystrophy, and other muscle-wasting disorders.
- Optimizing exercise strategies to enhance beneficial LIF signaling provides a translational basis for personalized exercise rehabilitation.

**Abstract:**

Leukemia inhibitory factor (LIF), a member of the interleukin-6 (IL-6) cytokine family, is a well-characterized myokine with pleiotropic regulatory effects on skeletal muscle. LIF modulates several fundamental cellular processes, including myoblast proliferation, apoptosis, angiogenesis, and energy metabolism. Exercise upregulates LIF expression in skeletal muscle, thereby promoting satellite cell activation, proliferation, myoblast differentiation, and angiogenesis, facilitating physiological muscle hypertrophy, and suppressing myocyte apoptosis and muscle atrophy. In addition, LIF plays a critical role in modulating the inflammatory and extracellular matrix remodeling following exercise-induced muscle damage, thereby supporting efficient muscle repair and regeneration. This review elaborates on the biological mechanisms by which LIF regulates skeletal muscle atrophy and contributes to the enhancement of skeletal muscle function. It also highlights the biological characteristics of myogenic LIF and discusses future directions for basic and applied research on exercise interventions targeting LIF signaling pathways.

## 1. Introduction

Acting dually as the primary effector organ for mammalian locomotion and a critical endocrine organ, skeletal muscle possesses a secretome that is intrinsically coupled to contractile activity and physical exercise [1]. It secretes diverse bioactive substances, including cytokines, regulatory peptides, adipokines, growth factors, and myokines, which regulate skeletal muscle growth, metabolism, and contractile function via paracrine or autocrine mechanisms [2]. Further research has revealed that skeletal muscle can also release myokines into the bloodstream via endocrine signaling, acting on cells of distant target organs to regulate systemic energy metabolism, oxidative stress, inflammatory responses, and endocrine homeostasis, thereby exerting crosstalk effects [1,3,4,5].

Myokines, also termed exercise factors, are defined as bioactive peptides endogenously synthesized and secreted by skeletal muscle in response to contractile activity [6,7]. Physiological muscle hypertrophy resulting from chronic exercise training is critically associated with a panel of skeletal muscle-secreted factors, including myokines, growth factors, and myostatin [1]. LIF, a myokine and member of the interleukin-6 cytokine family [8], exhibits pleiotropic biological activities and is endogenously expressed in almost all cell types and healthy tissues, including the heart and skeletal muscle. Divergent LIF mRNA isoforms, derived from the usage of alternative initiation codons, endow LIF with the capacity to mediate context-dependent autocrine and paracrine biological effects [9]. Exercise training robustly induces LIF expression in skeletal muscle, where it modulates myoblast proliferation, apoptosis, transcriptional regulation, angiogenesis, and energy metabolism [10]. Preclinical studies have established that LIF mitigates sarcopenia induced by aging or obesity, and that exercise confers protection against age-related metabolic diseases by improving mitochondrial dysfunction [11,12], Physiological muscle hypertrophy resulting from chronic exercise training is critically associated with a panel of skeletal muscle-secreted factors, including myokines, growth factors, and myostatin [13,14,15,16]. These findings collectively support a model in which exercise drives autocrine/paracrine LIF signaling in skeletal muscle to promote satellite cell activation, proliferation, and differentiation, inhibit myocyte apoptosis, and suppress muscle atrophy.

This review synthesizes current advances in the characterization of myogenic LIF and its modulation by exercise. We aim to delineate the core mechanisms by which LIF regulates skeletal muscle regeneration and mitigates muscle atrophy, highlight critical gaps in current research, and discuss the translational potential of LIF-targeted exercise interventions for the prevention and treatment of muscle atrophy, age-related sarcopenia, and sports injuries.

## 2. Molecular Structure of LIF and Its Receptor

LIF was initially identified as a factor mediating differentiation and repressing proliferation of the murine myeloid leukemia M1 cell line [9,17]. The human LIF gene localizes to chromosome 22 and the murine ortholog to chromosome 11; their protein-coding sequences are highly conserved across species (78–94% homology), whereas intronic and flanking regions exhibit greater divergence. LIF belongs to the long-chain helical bundle cytokine family [9,18]; the mature protein is generated via N-terminal cleavage of a precursor polypeptide, and native LIF is heavily glycosylated. Although the apparent molecular mass and structural properties of LIF are glycosylation-dependent, the deglycosylated core protein (harboring seven glycosylation sites and six cysteine residues) retains full biological activity. Intramolecular disulfide bonds are essential for maintaining LIF’s tertiary structure and function, and nuclear magnetic resonance analyses have confirmed that LIF is an exceptionally stable molecule [9,19].

The biological actions of LIF are entirely dependent on receptor-mediated intracellular signal transduction. The functional LIF receptor is a heterodimeric complex composed of a low-affinity, LIF-specific receptor subunit (LIFRβ, also known as gp190) and the common interleukin-6 signal transducer (IL6ST, universally known as gp130). LIFRβ is a single-pass transmembrane protein consisting of an N-terminal signal peptide and three core structural domains [20,21], and is widely expressed across most organs and cell types, mirroring the expression pattern of LIF. In addition to the membrane-bound form, a soluble LIFRβ isoform (sLIFR) exists, which inhibits LIF activity by sequestering extracellular LIF and preventing its binding to membrane receptors [9,20]. sLIFR can bidirectionally modulate the pro- and anti-inflammatory effects of LIF in a context-dependent manner, a regulatory mechanism analogous to the trans-signaling of the soluble IL-6 receptor (sIL-6R) [22]. As the common signal-transducing subunit for all IL-6 family cytokines, gp130 is a transmembrane glycoprotein with high binding affinity for LIF and is indispensable for downstream signal propagation. LIF, oncostatin M (OSM), cardiotrophin-1 (CT-1), and ciliary neurotrophic factor (CNTF) all initiate signaling via LIFRβ–gp130 heterodimers, with some cytokines requiring additional accessory subunits (e.g., CNTF requires CNTFRα). Notably, OSM can also signal via an alternative OSMR–gp130 complex [21,22,23]. A soluble gp130 isoform (sgp130) is endogenously present in human serum at physiological concentrations; it not only blocks IL-6/sIL-6R trans-signaling but also exerts antagonistic effects on the biological activities of CNTF, LIF, and OSM [22,24].

**Figure 1:**
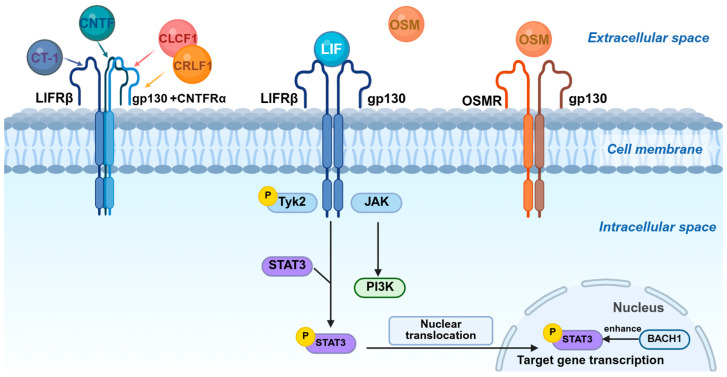
LIF receptor complex signaling in skeletal muscle. The functional LIF receptor is a heterodimer of LIFRβ and gp130. While LIF is the primary ligand, other IL-6 family cytokines can also signal via LIFR-containing complexes. LIF and OSM bind directly to LIFRβ, whereas CT-1, CNTF, and the CLCF1-CRLF1 heterodimer require the accessory CNTFRα subunit to initiate signaling. The CLCF1-CRLF1 complex competes with CNTF for binding to the CNTFR complex, activating the JAK–STAT signaling cascade. OSM can also signal via its specific OSMR–gp130 complex. BACH1 enhances STAT3 signaling and is shown here as a transcriptional regulator that recruits STAT3 to enhance signaling, as observed in stem cell models. Abbreviations: OSM, oncostatin M; CT-1, cardiotrophin-1; CNTF, ciliary neurotrophic factor; CLCF1, cardiotrophin-like cytokine factor 1; CRLF1, cytokine receptor-like factor 1; BACH1, BTB and CNC homology 1. The figure is created with BioRender.

The canonical LIF signal transduction cascade proceeds via a well-defined sequence: LIF first binds to LIFRβ, which subsequently recruits gp130 to form a high-affinity functional receptor complex. This complex triggers autophosphorylation and activation of receptor-associated Janus kinases (JAKs), including JAK1, JAK2, and TYK2, which are constitutively bound to the intracellular domains of LIFRβ and gp130 [19,22]. JAK1 is considered the primary kinase mediating LIF signaling, which in turn activates three core downstream cascades: the JAK/signal transducer and activator of transcription 3 (STAT3), mitogen-activated protein kinase (MAPK)/extracellular signal-regulated kinase (ERK), and phosphoinositide 3-kinase (PI3K)/Akt pathways [9]. BTB and CNC homology 1 (BACH1), a transcription factor that functions as a transcriptional repressor of oxidative stress response genes and heme oxygenase-1 (HO-1) [25], recruits STAT3 to enhance LIFR signaling activity, further augmenting the self-renewal capacity of mouse embryonic stem cells and refining the regulatory network of the LIF/LIFR/STAT3 axis [26] (Figure 1).

## 3. Physiological and Pathophysiological Roles of LIF

**Figure 2:**
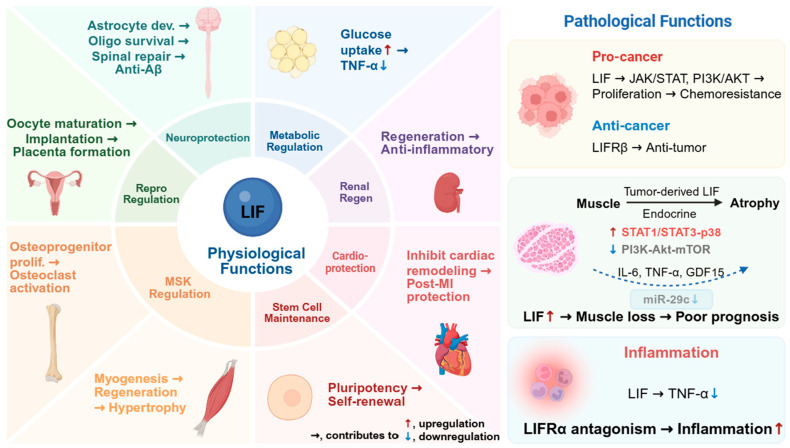
Physiological and pathophysiological roles of LIF in mammalian organ systems. Under physiological conditions, LIF mediates neuroprotection, reproductive regulation (Repro Regulation), musculoskeletal homeostasis, stem cell pluripotency, cardioprotection, renal regeneration (Renal Regen), and systemic metabolic regulation. In pathological contexts, LIF exhibits dual pro- and anti-tumorigenic functions: pro-tumorigenic LIF activates JAK/STAT and PI3K/AKT signaling to promote proliferation and chemoresistance, whereas LIFRβ mediates tumor-suppressive effects. Tumor-derived LIF drives cancer cachexia through pro-catabolic STAT3-p38 MAPK signaling, elevating TNF-α and inducing progressive muscle atrophy and poor prognosis. LIFRβ antagonism exacerbates inflammation and impairs myotube formation. ↑, upregulation; ↓, downregulation; →, contributes to. Abbreviations: LIF, leukemia inhibitory factor; JAK, Janus kinase; STAT, signal transducer and activator of transcription; PI3K, phosphoinositide 3-kinase; AKT, protein kinase B; LIFRβ, LIF receptor β; TNF-α, tumor necrosis factor-α. The figure is created with BioRender.

While initially characterized for its ability to induce myeloid leukemia cell differentiation and inhibit proliferation [27], subsequent studies have established LIF as a pleiotropic cytokine that regulates diverse physiological and pathological processes across mammalian tissues [28]. LIF target genes are expressed in most cell types, including embryonic and hematopoietic stem cells, neurons, immune cells, hepatocytes, and osteoblasts/osteoclasts, where LIF modulates core cellular processes, including proliferation, apoptosis, transcriptional regulation, embryonic lineage specification, and stem cell pluripotency maintenance [29]. LIF also participates in tissue regeneration in the nervous system, skeletal muscle, kidney, liver, and bone [10,30,31], making it indispensable for stem cell homeostasis and tissue repair (Figure 2).

In the nervous system, LIF crosses the blood–brain barrier and mediates astrocyte development [32], oligodendrocyte survival, and spinal cord repair following injury [33]. In retinal pathology, LIF is upregulated in glial cells as an endogenous cytoprotective mechanism [17,34]. LIF and its receptor regulate hypothalamic–pituitary–adrenal axis activation and pituitary development [9]. LIF mitigates amyloid-β neurotoxicity via Akt/ERK-mediated c-fos induction, representing a potential therapeutic target for Alzheimer’s disease [33]. LIF mRNA is highly expressed in embryonic murine muscle at E17 (the stage of secondary myogenesis completion) and declines rapidly postnatally. Recombinant LIF delays neuromuscular synapse elimination in neonates, while LIF-deficient mice show reduced postsynaptic motor endplate area [35]. LIFR colocalizes with neuromuscular junctions, implicating a role in muscle–nerve synaptic signaling. In the reproductive system, LIF promotes porcine oocyte maturation via the LIF/LIFR/STAT3 signaling pathway [36,37], and regulates endometrial decidualization and blastocyst implantation, consistent with high LIF expression in the uterus, ovaries, and fallopian tubes during early pregnancy [37]. LIF exerts a non-redundant role in maternal blastocyst receptivity, placentation, and embryonic neural development. In stem cell biology, LIF has practical applications in maintaining the self-renewal capacity and pluripotency of embryonic stem cells and induced pluripotent stem cells; mouse embryonic stem cell viability decreases in a dose-dependent manner at 72 h after radiation exposure, whereas LIF significantly improves cell viability [38,39]. In the musculoskeletal system, LIF modulates bone resorption, formation, and metabolism, as well as skeletal muscle function. In bone tissue, LIF promotes osteoprogenitor proliferation by binding to osteoblastic LIFR. Although LIFR expression has not been detected on osteoclast membranes, LIF indirectly activates osteoclasts by modulating osteoblast activity, thereby promoting osteoclastic bone resorption [40,41,42]. Osteoporosis and rheumatoid arthritis involve functional imbalance between osteoblasts and osteoclasts; LIF overexpression drives mesenchymal stem cell differentiation toward the osteoclast lineage, accelerating post-injury bone repair. Mechanical stress induces LIF expression in bone cells and regulates osteoclast/osteoblast formation via enhanced STAT3 phosphorylation [40,43]. In skeletal muscle, therapeutic LIF gene transfer ameliorates muscle atrophy in dystrophic mice via immune modulation [44,45], whereas myeloid cell-mediated targeting of LIF to dystrophic muscle triggers transient increases in muscle fiber lesions [45]. LIF enhances myoblast survival, positively regulates myogenesis [46], and promotes muscle regeneration and hypertrophy [8,38]. LIF regulates cardiomyocyte hypertrophy, energy metabolism, cardiac remodeling, and cardioprotection; after myocardial infarction, LIF is secreted in cardiac tissue, and LIF preconditioning improves cardiac function, reduces infarct size, and inhibits remodeling [17]. LIF is closely associated with post-injury kidney regeneration and inflammatory responses, promoting differentiation of embryonic renal mesenchymal and epithelial cells while enhancing the regenerative capacity of renal tubular epithelial cells [10,47]. As a multifunctional protein, LIF participates in adipocyte metabolism and systemic energy homeostasis regulation; via autocrine/paracrine mechanisms, it increases muscle glucose uptake and inhibits adipocyte differentiation [48,49]. LIF possesses well-defined anti-inflammatory properties [50,51], downregulating lipopolysaccharide-induced TNF-α expression, whereas LIFRβ antagonism during muscle regeneration exacerbates inflammation and inhibits myotube formation [52]. In cancer biology, the LIF/LIFR axis exerts pleiotropic effects, exhibiting both pro-tumorigenic and tumor-suppressive functions with respect to cancer cell proliferation, migration, and invasion [17,20,53,54]. LIF is overexpressed in solid tumors such as breast, prostate, lung, gastric, and pancreatic cancers [55,56,57]. and promotes tumor progression via JAK/STAT and PI3K/AKT pathways; LIF inhibition impairs cancer cell proliferation, adhesion, migration, and colony formation. Conversely, LIFRβ may exert anti-tumor effects through activation of tumor suppressor-associated signaling pathways [20,58,59].

Beyond its tissue-specific roles, exercise recruits LIF as a systemic inter-organ signal that coordinates adaptive responses across multiple organ systems. As an exercise-responsive myokine, LIF is released from contracting skeletal muscle into the circulation, enabling it to reach remote target organs and exert effects that cannot be replicated by local paracrine signaling alone. Most notably, circulating exercise-induced LIF reaches the myocardium, where it synergizes with exercise training to improve cardiac function, reduce infarct size, and confer cardioprotection [60], providing a mechanistic basis for the well-established cardiovascular benefits of physical activity. This muscle-heart axis exemplifies a broader paradigm in which skeletal muscle acts as an endocrine organ during exercise, mobilizing LIF as a humoral mediator of systemic physiological adaptation.

LIF exerts diametrically opposed effects on skeletal muscle mass in physiological versus pathological contexts, a functional duality defined by its cellular source, signaling mode, and tissue microenvironment. Under physiological conditions such as resistance exercise or muscle injury, myogenic LIF is secreted by activated myotubes and satellite cells and acts via local autocrine/paracrine signaling to promote muscle hypertrophy and regeneration. In contrast, tumor-derived LIF drives cancer cachexia, a lethal syndrome characterized by progressive muscle wasting, metabolic dysregulation, and poor prognosis [53,61]. Mechanistically, tumor-derived LIF acts systemically via endocrine signaling to induce muscle atrophy through selective activation of pro-degradative STAT1/STAT3–p38 MAPK signaling, without engaging the anabolic PI3K/Akt/mTOR cascade activated by myogenic LIF [62]. This pro-atrophic effect is further amplified by synergistic crosstalk with other cachectic factors, including IL-6, TNF-α, and growth differentiation factor 15 (GDF15), which collectively form a feedforward pro-inflammatory network that accelerates muscle protein breakdown [59]. For example, LIF-induced STAT3 activation upregulates IL-6, which in turn enhances LIF-mediated signaling in myotubes [63]. LIF modulates microRNAs such as miR-29c; its downregulation in lung cancer removes suppression of pro-atrophic genes and accelerates muscle wasting [64]. Clinically, elevated serum LIF correlates with muscle loss, poor treatment response, and shortened survival in metastatic cancer, establishing LIF as a predictive biomarker and therapeutic target for cancer cachexia [65,66]. In summary, continued investigation into the biological functions of LIF holds significant theoretical and clinical implications for the prevention and treatment of numerous diseases.

## 4. Myogenic LIF in Skeletal Muscle: Functional Roles

### 4.1. Induction and Regulation of Myogenic LIF

As the primary effector of locomotion, skeletal muscle is both a target of endocrine regulation and a key endocrine organ, mediating inter-organ communication via the secretion of bioactive molecules [67]. Under resting conditions, LIF mRNA expression in skeletal muscle is barely detectable; however, diverse stressors-including inflammation, muscle injury, and contractile activity from exercise-drive robust transcriptional upregulation and protein secretion of LIF [68]. Muscle injury induces a rapid and transient upregulation of LIF expression, which mitigates myofiber degeneration and provides key regulatory signals for muscle regeneration [69]. Mechanical stimuli from overload or resistance exercise similarly drive robust LIF synthesis and secretion in skeletal muscle, supporting a role for LIF as a contraction-induced myokine that mediates adaptive muscle hypertrophy via autocrine/paracrine signaling [70,71]. In vitro studies confirm that electrical stimulation (a model of muscle contraction) significantly upregulates LIF expression in primary human myotubes, while in vivo studies demonstrate that resistance training markedly induces LIF expression in human skeletal muscle [72]. Mechanistically, LIF mRNA expression in skeletal muscle is transcriptionally regulated by AMP-activated protein kinase (AMPK) via modulation of LIF gene promoter activity, rather than alterations in mRNA stability [73].

While myotubes are the primary source of exercise-induced LIF, other cell types in the muscle microenvironment—including infiltrating mononuclear cells (macrophages and neutrophils), neurons, and Schwann cells in peripheral nerves—also secrete LIF in the setting of muscle injury. Collectively, muscle damage and contractile activity are the two primary triggers of myogenic LIF expression and secretion [69].

### 4.2. LIF in Skeletal Muscle Hypertrophy and Regeneration

LIF is a well-established contraction-induced myokine that promotes physiological skeletal muscle hypertrophy and mitigates muscle atrophy by enhancing muscle regeneration and inhibiting myocyte apoptosis. In a preclinical rat model of MI-induced skeletal muscle atrophy, 8 weeks of interval exercise training significantly upregulated LIF and LIFR expression in the gastrocnemius muscle and activated STAT3 phosphorylation, which coincided with inhibited myocyte apoptosis, attenuated skeletal muscle atrophy, and improved muscle contractile function [74]. This finding provides in vivo correlative evidence supporting an association between exercise-mediated LIF upregulation and muscle mass preservation in disease states, suggesting that LIF signaling may contribute to the protective effects of exercise against pathological skeletal muscle atrophy. However, as this study was not specifically designed to isolate or modulate LIF signaling, a direct causal relationship remains to be established through targeted loss-of-function or gain-of-function approaches. The non-redundant role of LIF in muscle hypertrophy is further corroborated by genetic loss-of-function studies, wherein LIF-knockout mice exhibit blunted and delayed hypertrophic responses to mechanical overload [59]. LIF exerts fiber type-specific effects on skeletal muscle: four weeks of systemic LIF administration induces hypertrophy exclusively in the slow-twitch soleus muscle, while hypertrophy of the fast-twitch extensor digitorum longus requires synergistic signaling with β_2_-adrenoceptor agonists, a difference that aligns with the divergent metabolic and contractile properties of type I and type II myofibers [75]. At the cellular level, LIF enhances myoblast survival and inhibits caspase-dependent apoptosis, while its effects on myoblast proliferation are context-dependent, with studies reporting divergent effects on mitotic rate [28]. In vivo, daily LIF administration for four weeks increases soleus muscle mass and contractile force in healthy adult rats and exerts synergistic hypertrophic effects on both fiber types when combined with the β_2_-agonist clenbuterol [44]. During muscle regeneration, LIF exhibits a dynamic expression pattern: in contused rat gastrocnemius, LIF mRNA is rapidly and transiently upregulated within 3–12 h post-injury; in notexin-injured mouse tibialis anterior, LIF is biphasically upregulated, with the first peak coinciding with pro-inflammatory cytokines and proliferation markers, and the second peak correlating with myogenic differentiation and myotube formation [76].

The pro-regenerative and pro-hypertrophic effects of LIF are mediated by three core mechanisms: anti-inflammatory signaling, satellite cell pool regulation, and modulation of myogenic differentiation [35]. At the inflammatory level, LIF exerts intrinsic anti-inflammatory effects during the early phase of muscle regeneration by inhibiting neutrophil infiltration and suppressing the expression of pro-inflammatory cytokines including TNF-α, IL-1β, and IL-6. Blockade of LIFR signaling exacerbates local pro-inflammatory responses and delays myofiber regeneration, while LIF administration protects dystrophic myofibers from necrosis via attenuation of inflammatory signaling [77]. At the cellular level, LIF is a potent regulator of satellite cells, the resident muscle stem cells indispensable for hypertrophy and regeneration. Quiescent under homeostatic conditions, satellite cells are activated by muscle injury or exercise-induced mechanical stress to drive de novo myofibril formation. LIF potently stimulates satellite cell proliferation after injury, a role initially demonstrated through the identification of multiple signaling pathways mediating LIF-induced satellite cell proliferation, including JAK2-STAT3 [78]. Satellite cell-derived LIF signaling is also required for muscle regrowth following atrophy [79]. Consistent with these findings, LIF accelerates muscle regeneration, enhances the expansion of transplanted satellite cells, and upregulates the expression of proliferation-associated transcription factors JunB and c-Myc in myoblasts via autocrine activation of the JAK2–STAT3–PI3K pathway [80,81]. At the molecular level, LIF balances myoblast proliferation and differentiation to optimize muscle regeneration. LIF inhibits caspase-3 activation to repress premature myogenic differentiation of muscle progenitor cells, thereby preserving the proliferative capacity of the muscle stem cell pool. While LIF does not directly increase basal myoblast proliferation rates, it prevents the time-dependent decline in proliferative capacity, ensuring a sufficient myoblast pool for tissue repair. LIF suppresses the myogenic transcription factors MyoD and myogenin under differentiation conditions, maintaining the proliferative myoblast population and coordinating the balance between proliferation and differentiation during regeneration [76,80]. Together, these mechanisms underpin the orchestrated regulation of inflammatory resolution and myogenic reconstruction that defines successful skeletal muscle repair.

LIF exhibits functional duality in skeletal muscle, promoting hypertrophy under physiological conditions yet inducing atrophy in pathological states. This divergence stems from context-dependent differences in secretion source, signaling patterns, and the tissue microenvironment. Under physiological conditions such as resistance exercise or muscle contusion, myogenic LIF is secreted by activated myotubes and satellite cells and acts via autocrine/paracrine pathways to coordinate adaptive muscle remodeling [82]. JAK2–STAT3 signaling drives satellite cell proliferation and preserves the stem cell pool via inhibition of caspase-3-mediated apoptosis, while PI3K–Akt–mTOR signaling enhances de novo muscle protein synthesis. LIF’s anti-inflammatory activity further supports muscle homeostasis by suppressing excessive pro-inflammatory cytokine production and limiting tissue damage. This anabolic signaling exhibits fiber type specificity, with slow-twitch (type I) fibers showing preferential hypertrophy in response to myogenic LIF, driven by higher basal LIFRβ expression [35,83]. In pathological contexts such as cancer cachexia, this regulatory balance is completely disrupted. Tumor-derived LIF, secreted into the systemic circulation, acts via endocrine signaling on skeletal muscle to trigger selective activation of pro-degradative pathways—JAK/STAT1/3 and p38 MAPK—without engaging anabolic PI3K/Akt/mTOR cascades [64]. This uncoupling of anabolic and catabolic signaling is linked to altered receptor dynamics: elevated soluble LIFR (sLIFR) in cachectic serum may interfere with membrane-bound LIFRβ–gp130 complex formation, shifting signaling toward pro-degradative outputs [84]. LIF synergizes with other cachectic mediators such as GDF15 and myostatin to amplify muscle protein breakdown: GDF15 upregulates LIF expression via ERK1/2–c-Fos signaling, while LIF reciprocally enhances myostatin-mediated inhibition of myogenesis [85]. Fiber type specificity persists in this pathological context, with slow-twitch fibers exhibiting greater susceptibility to LIF-induced atrophy due to their higher metabolic sensitivity to pro-inflammatory signals. Epigenetic mechanisms further modulate LIF’s functional switch: in cancer cachexia, tumor-derived inflammatory cues induce hypomethylation of the LIF promoter, enhancing its transcriptional activity in skeletal muscle, whereas exercise induces DNA methylation of Nr4a3—a canonical exercise-responsive gene and key upstream regulator of LIF—to promote anabolic signaling, highlighting the role of epigenetic reprogramming in shaping LIF’s context-dependent biological effects in skeletal muscle.

### 4.3. LIF-Mediated Signaling Pathways in Skeletal Muscle

Upon binding to the LIFRβ–gp130 heterodimer, LIF activates three core, non-redundant intracellular signaling pathways—JAK2–STAT3, PI3K–Akt1–mTOR, and MEK–ERK1/2—that drive skeletal muscle hypertrophy. Structural studies have identified conserved motifs in the intracellular domains of gp130 and LIFRβ that mediate recruitment and activation of SH2 domain-containing molecules (STAT3, STAT1, SHP2) [19].

#### 4.3.1. JAK2-STAT3 Pathway

Among the STAT family members (STAT1, STAT3, STAT5) activated by gp130 family cytokines, STAT3 is the primary mediator of LIF’s cellular effects in skeletal muscle. LIFR is expressed on both satellite cells and myoblasts, and LIF binding triggers JAK2-mediated phosphorylation of STAT3, which promotes STAT3 dimerization and nuclear translocation. Nuclear STAT3 induces transcription of downstream target genes, including proliferation-associated factors JunB and c-Myc and anti-apoptotic factors, thereby regulating myoblast proliferation, survival, and apoptosis. Activated STAT3 also induces the expression of suppressor of cytokine signaling 3 (SOCS3), which forms a negative feedback loop to terminate JAK/STAT signaling via binding to JAK1 and gp130 or direct inhibition of JAK2 kinase activity. The JAK2–STAT3 pathway is widely accepted as the core signaling cascade mediating LIF’s pro-hypertrophic and pro-regenerative effects in skeletal muscle [86,87].

#### 4.3.2. PI3K-Akt1-mTOR Pathway

The PI3K–Akt1–mTOR cascade is the master regulator of muscle protein synthesis and is indispensable for LIF-induced skeletal muscle hypertrophy. PI3K, Akt1, and mTOR all regulate LIF release, with chemical inhibition of any of these molecules significantly downregulating LIF expression. Electrical stimulation and mechanical overload robustly promote LIF secretion, which in turn drives muscle hypertrophy via the PI3K/Akt/mTORC1 axis. In vitro studies confirm that LIF acutely induces protein synthesis in myotubes, concomitant with activation of both STAT3 and the Akt–mTORC1 pathway; gp130 knockout or Akt inhibition completely abrogates LIF-induced protein synthesis. Notably, LIF can also induce myotube protein synthesis via mTORC1-independent mechanisms, while LIF-induced SOCS3 exerts negative feedback regulation on this anabolic cascade [88,89].

#### 4.3.3. MEK-ERK1/2 Pathway

**Figure 3:**
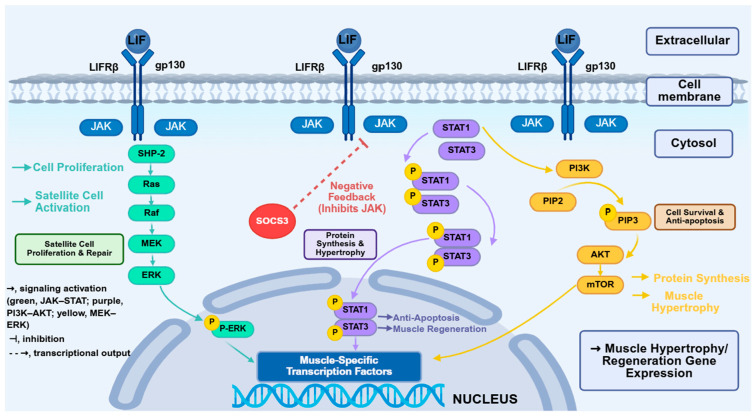
Core signaling pathways mediating LIF’s regulation of skeletal muscle cell proliferation and apoptosis. LIF binding to the LIFRβ/gp130 complex activates three parallel cascades—JAK/STAT (green), PI3K-AKT (purple), and MEK-ERK (yellow)—converging on nuclear transcription factors to drive specific myogenic and hypertrophic gene expression programs. Rather than acting in parallel with equal intensity, the activation of these pathways is context-dependent and differentially prioritized to fulfill distinct physiological requirements: satellite cell proliferation and repair (green), protein synthesis and hypertrophy (purple), and cell survival and anti-apoptosis (yellow). SOCS3 forms a negative feedback loop inhibiting JAK/STAT signaling and suppresses MEK–ERK1/2 by displacing SHP2 from LIFR binding sites. →, signaling activation; ⊣, inhibition; - - →, transcriptional output. Abbreviations: LIFRβ, LIF receptor β; gp130, glycoprotein 130; JAK, Janus kinase; STAT3, signal transducer and activator of transcription 3; SOCS3, suppressor of cytokine signaling 3; SHP2, Src homology-2 domain-containing phosphatase 2; PI3K, phosphoinositide 3-kinase; AKT, protein kinase B; mTOR, mechanistic target of rapamycin; MEK, mitogen-activated protein kinase kinase; ERK, extracellular signal-regulated kinase. The figure is created with BioRender.

The MEK–ERK1/2 pathway mediates LIF’s inhibitory effects on premature myogenic differentiation, thereby preserving the muscle stem cell pool during regeneration. LIF inhibits caspase-3 activation and myoblast differentiation via MEK/ERK signaling; caspase-3 activity is essential for normal myogenic differentiation, and LIF-mediated ERK activation suppresses this process to prevent premature differentiation of myogenic progenitors. Multiple IL-6 family cytokines downstream of LIFR, including CT-1, OSM, and CNTF, similarly inhibit myogenic differentiation via MEK/ERK-dependent mechanisms. Beyond differentiation, the ERK1/2 pathway also promotes muscle protein synthesis in response to diverse hypertrophic stimuli and participates in the repair of glucocorticoid-induced muscle atrophy. Collectively, while all three pathways are activated upon LIF binding, the JAK2-STAT3 pathway is established as the core signaling cascade because it serves as the primary mediator of fundamental cellular behaviors such as myoblast proliferation, survival, and anti-apoptosis by directly regulating nuclear transcription factors including JunB and c-Myc [86,87]. The other two pathways execute specialized, non-redundant functions. The PI3K-Akt1-mTOR pathway acts as the master regulator of de novo muscle protein synthesis, a process that is completely abrogated if this specific axis is inhibited [89,90]. Meanwhile, the MEK-ERK1/2 pathway provides critical temporal control by preventing premature myogenic differentiation, thereby preserving the muscle stem cell pool during regeneration [88]. The relative contribution of each pathway is highly context-dependent. In physiological hypertrophy, they work in concert, whereas in pathological states such as cancer cachexia, the uncoupling of these signals, in which pro-degradative STAT3 signaling is activated without the compensatory anabolic PI3K-Akt-mTOR cascade, drives progressive muscle wasting [62,64] (Figure 3).

## 5. Exercise-Induced LIF in Skeletal Muscle

### 5.1. LIF-Mediated Skeletal Muscle Adaptation

Exercise training drives the secretion of a diverse array of myokines from skeletal muscle, including LIF, IL-6, irisin, IGF-1, IL-8, IL-15, myostatin, and musclin, which collectively promote skeletal muscle growth, hypertrophy, and angiogenesis while exerting systemic endocrine effects on adipose tissue, the nervous system, liver, and brain [67,89]. Among these, LIF has emerged as a key exercise-responsive myokine, with its secretion tightly modulated by exercise modality, intensity, duration, and training status. Chronic exercise training induces skeletal muscle hypertrophy in concert with a robust upregulation of LIF at both the gene and protein levels. Evidence from human resistance training interventions and primary myotube culture models demonstrates that LIF functions as a contraction-induced myokine—promoting satellite cell proliferation through autocrine and paracrine signaling mechanisms [91,92]. The magnitude and nature of exercise-induced LIF responses vary substantially across training modalities, revealing complex regulatory mechanisms. Aerobic exercise upregulates LIF mRNA in human skeletal muscle, yet LIF protein levels decline rapidly after exercise cessation despite sustained transcriptional elevation, indicating that aerobic exercise primarily drives LIF transcription without robustly promoting translation [93]. Consistent with this, acute exhaustive exercise produces a 9-fold increase in skeletal muscle LIF mRNA at 6 h post-exercise followed by gradual decline, with no significant change in LIF protein and no detectable plasma LIF [25,27]. This mRNA-protein dissociation is partly attributable to the inherent instability of LIF mRNA, which contains adenylate-uridylate-rich elements (AREs) in its 3′-untranslated region that confer a half-life of approximately 20–30 min [94]. LIF mRNA turnover is governed by competing RNA-binding proteins: tristetraprolin (TTP/ZFP36) promotes rapid ARE-mediated mRNA decay via recruitment of the CCR4-NOT deadenylase complex [95], whereas the mRNA-stabilizing factor HuR (ELAVL1) may antagonize TTP-mediated LIF mRNA decay, consistent with their established competition for ARE motifs in other mRNA targets [96]. The transient nature of exercise-induced signaling may be insufficient to durably shift this balance toward mRNA stabilization, thereby limiting LIF protein accumulation despite robust transcriptional induction. In contrast, interval exercise training in rats significantly upregulates both LIF and LIFR expression in the gastrocnemius muscle, activating STAT3 phosphorylation to attenuate skeletal muscle apoptosis and atrophy [74]. Progressive resistance training robustly upregulates myogenic LIF expression in human skeletal muscle, with post-exercise changes in LIF levels correlating significantly with expression of matrix metalloproteinase 14 (MMP14), a key regulator of extracellular matrix remodeling [89]. In vitro electrical pulse stimulation studies-used as a model of exercise-further confirm that 24 h of stimulation significantly increases LIF concentrations in the conditioned medium of primary human myotubes [97]. Exercise duration and intensity exert dose-dependent effects on LIF secretion that are further modulated by training status. In a mouse treadmill training study, long-duration exercise (55 min/day) increased plasma LIF levels by 33% within 48–72 h, whereas short-duration exercise (10 min/day) had no significant effect [98]. Training status also shapes the LIF response: in a study comparing elite athletes and untrained volunteers, static isometric exercise increased plasma LIF levels by approximately 50%, while dynamic cycle exercise had no significant effect, with plasma LIF returning to baseline within 30 min post-exercise in all groups except untrained volunteers performing static exercise [72]. Chronic resistance training further modulates resting systemic myokine profiles in an age-dependent manner—after 12 weeks of resistance exercise, resting irisin concentrations are lower in older males than in younger males, while apelin and IL-15 concentrations are increased across the full cohort [99]. These modality- and dose-specific differences likely reflect divergent patterns of muscle fiber type recruitment, differential activation of intracellular signaling cascades, and modality-specific excitation–transcription coupling mechanisms. Resistance exercise preferentially recruits fast-twitch type IIa fibers, which exhibit the most pronounced transcriptomic responses to mechanical loading, and involves a high proportion of eccentric contractions—a well-established stimulus for LIF upregulation [100]. Mechanistically, intracellular Ca^2+^ oscillations mediate exercise-induced LIF upregulation, as stimulation of muscle cells with a Ca^2+^ onophore significantly increases LIF expression; exercise-induced muscle hypoxia represents an additional regulatory mechanism, as hypoxic conditions independently stimulate LIF secretion from myotubes [93]. These findings are summarized in Table 1, which synthesizes current evidence on the effects of different exercise modalities on LIF expression. These findings are summarized in Table 1, which synthesizes current evidence on the effects of different exercise modalities on LIF expression.

Beyond its autocrine/paracrine actions within skeletal muscle, exercise-induced LIF is released into circulation as an endocrine factor mediating systemic cross-organ communication. LIF secreted by skeletal muscle post-exercise targets adipose and hepatic tissues to regulate systemic glucose and lipid metabolism, and, acting in concert with other myokines such as IL-6 and irisin, functions as an anti-inflammatory mediator that reduces chronic low-grade inflammation [8,102]. Cytokines of the LIFR family-including LIF and OSM-mediate systemic health benefits of exercise by acting on peripheral target organs; for example, OSM in the serum of exercised mice significantly inhibits breast cancer cell proliferation [83]. Exercise-induced LIF also acts as a key mediator of the skeletal muscle–cardiac axis, exerting cardiovascular protection through regulation of vascular inflammation, coagulation, and angiogenesis [50]. These systemic actions carry direct therapeutic relevance: exercise serves as a first-line intervention for sarcopenic obesity, a prevalent clinical disorder characterized by concurrent skeletal muscle loss and adipose tissue dysfunction, in which myokines including LIF and adipokines collectively restore muscle mass, alleviate chronic inflammation, and rebalance systemic metabolic homeostasis [102]. Supporting this, mice allowed free-wheel running for two weeks exhibit significantly elevated LIF gene expression in the tibialis anterior and diaphragm muscles, with this upregulation exerting anti-inflammatory effects and promoting the survival of transplanted myoblasts in dystrophic rats [44]. Notably, non-weighted endurance running is a weaker hypertrophic stimulus than resistance exercise [103]. Thus, LIF upregulation during running may primarily reflect roles in muscle homeostasis and protein turnover rather than hypertrophy. Consistent with this, the strongest human evidence linking LIF to muscle hypertrophy derives from resistance training, where progressive loading involving greater mechanical strain and eccentric muscle damage robustly upregulates myogenic LIF [104]. Whether running-induced LIF elevation contributes meaningfully to human hypertrophy remains to be established in studies that directly modulate LIF signaling.

**Figure 4:**
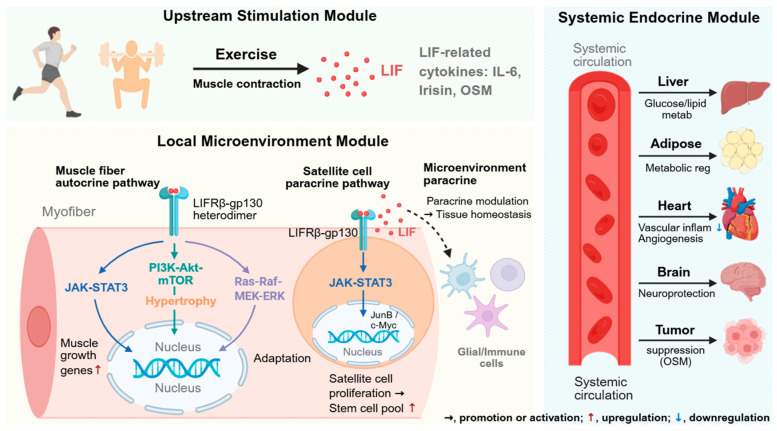
LIF-mediated autocrine/paracrine and endocrine signaling in exercise-induced skeletal muscle adaptation. Exercise-induced muscle contraction stimulates LIF secretion, cooperatively with IL-6, IL-4, and OSM. LIF acts on myofibers via autocrine signaling and on satellite cells via paracrine signaling through the LIFRβ/gp130 receptor complex, activating JAK/STAT, PI3K–Akt–mTOR, and Ras–Raf–MEK–ERK cascades to drive muscle gene expression, satellite cell proliferation, and microenvironmental homeostasis. Systemically, circulating LIF targets the liver, adipose tissue, heart, and brain to regulate metabolism, cardioprotection, and neuroprotection. →, activation or upregulation; ⊣, inhibition or downregulation. Abbreviations: LIF, leukemia inhibitory factor; IL-6, interleukin-6; OSM, oncostatin M; LIFRβ, LIF receptor β; gp130, glycoprotein 130; JAK, Janus kinase; STAT, signal transducer and activator of transcription; PI3K, phosphoinositide 3-kinase; Akt, protein kinase B; mTOR, mechanistic target of rapamycin; MEK, mitogen-activated protein kinase kinase; ERK, extracellular signal-regulated kinase. The figure is created with BioRender.

At the molecular level, exercise-induced myogenic LIF acts via autocrine/paracrine signaling through LIFRβ–gp130 receptor complexes on myofibers and satellite cells, activating the JAK–STAT3, PI3K–Akt–mTOR, and Ras–Raf–MEK–ERK pathways. In myofibers, these cascades converge to upregulate muscle growth-associated genes, driving physiological hypertrophy and functional adaptation. In satellite cells, JAK–STAT3 signaling upregulates the proliferation-associated transcription factors JunB and c-Myc to promote expansion of the stem cell pool. LIF also modulates glial and immune cell activity in the muscle microenvironment via paracrine signaling to maintain tissue homeostasis, collectively orchestrating the adaptive and regenerative responses of skeletal muscle to exercise (Figure 4).

### 5.2. Epigenetic Regulation of Exercise-Induced LIF Expression

DNA methylation changes at the LIF promoter may represent one mechanism through which contractile activity influences LIF transcription. In skeletal muscle, the LIF promoter contains methylation-sensitive CpG sites that are dynamically regulated in response to mechanical loading, and demethylation of these sites correlates with increased LIF mRNA output following acute exercise [105]. Genome-wide bisulfite sequencing in exercised muscle has further revealed that exercise-induced hypomethylation is enriched at enhancer regions harboring NF-κB and AP-1 binding motifs, transcription factors known to drive LIF transcription in contracting myofibers [105,106]. However, direct evidence causally linking exercise-induced methylation changes at the LIF locus to its transcriptional activation remains limited.

In addition to DNA methylation, histone modifications constitute a second layer of epigenetic regulation over LIF expression. Exercise promotes the nuclear export of HDAC4 and HDAC5 in skeletal muscle, thereby relieving MEF2-dependent transcriptional repression [107]. Electrical pulse stimulation of cultured myotubes is sufficient to induce LIF transcription [104]. Given that pharmacological HDAC inhibition can recapitulate certain exercise-like transcriptional and metabolic adaptations [108,109], it will be important to determine whether LIF is likewise subject to HDAC-mediated repression in resting skeletal muscle. Critically, whether MEF2 directly mediates this effect by binding the LIF proximal promoter remains to be determined.

Non-coding RNAs may confer an additional layer of epigenetic regulation over LIF expression. The long non-coding RNA MALAT1 is downregulated by acute endurance exercise [110]. In skeletal muscle, MALAT1 interacts with the PRC2 complex to mediate H3K27me3 deposition at target gene loci [111]. Whether MALAT1 similarly recruits PRC2 to the LIF locus during muscle regeneration, however, remains unknown. Reciprocally, miR-1 and miR-206 are both downregulated by endurance training [112]. These microRNAs are predicted to target the 3′ untranslated region of LIF mRNA [113,114], implicating a convergent non-coding RNA network in the control of LIF expression. Their exercise-induced suppression may therefore relieve miRNA-mediated degradation of LIF mRNA, potentially contributing to the stabilization of LIF transcript and protein in skeletal muscle. Collectively, these findings suggest a multilayered epigenetic program converging on the LIF locus to regulate its expression in response to exercise. Direct experimental evidence of exercise-induced epigenetic modifications specifically at the LIF locus, however, remains limited. Understanding these regulatory mechanisms may offer therapeutic targets for conditions in which exercise-induced LIF signaling is impaired, such as age-related sarcopenia or disuse atrophy.

### 5.3. Exercise-Induced Proteomic Remodeling and LIF Signaling

Distinct exercise modalities exert divergent effects on LIF and its downstream protein networks. PoWeR training simultaneously upregulates LIF and multiple muscle health-associated proteins, including LACTB, MIB1, and UBR4. Mechanistically, LACTB maintains myocyte metabolic homeostasis via regulation of mitochondrial membrane structure; MIB1 preserves type II muscle fiber diversity and satellite cell homeostasis; and UBR4 mediates muscle protein quality control. These proteins act synergistically with LIF to improve skeletal muscle function in aged mice [73]. Aging modulates the skeletal muscle proteomic response to exercise: compared to young mice (4–6 months old), aged mice (22–24 months old) subjected to identical weighted wheel running exhibit only approximately 30% overlap in differentially expressed skeletal muscle proteins (e.g., NADH dehydrogenase-related proteins) [73]. Proteins specifically upregulated in aged mice are predominantly enriched in ribosomal pathways, whereas those in young mice are more closely associated with energy metabolism-related cascades. Given that ribosomal proteins are key effectors within LIF downstream signaling networks [115], these age-dependent proteomic differences may reflect differential reliance on protein synthesis versus energy metabolism pathways during exercise adaptation. However, whether LIF directly drives this age-specific proteomic divergence remains to be determined. Moreover, the upstream triggers that shift aged skeletal muscle toward this greater reliance on LIF-mediated pathways remain unknown. Whether this shift originates from age-related changes in LIFRβ abundance, STAT3 sensitivity, or specific epigenetic reprogramming requires further dissection. Exercise duration also modulates LIF-associated proteomic remodeling: long-term treadmill training (55 min/day) significantly upregulates VPS13A (positively correlated with muscle mass) and NPL (associated with muscle fatigue resistance), suggesting that LIF may mediate duration-dependent improvements in muscle function via epigenetic regulation of these proteins. These proteomic differences highlight the modality-specific nature of exercise-mediated LIF regulation, providing a molecular basis for optimizing LIF-targeted exercise interventions [116,117,118]. An important but understudied question concerns whether the metabolism and clearance of secreted LIF are altered with aging. Age-related changes in hepatic and renal function, systemic inflammation, and receptor-mediated endocytosis could potentially affect LIF half-life and bioavailability in older individuals. If LIF is metabolized more slowly in aged hosts, this could result in prolonged signaling duration and potentially altered downstream effects. Conversely, reduced receptor sensitivity could necessitate higher LIF concentrations for equivalent biological activity. These pharmacokinetic considerations represent a significant gap in our understanding of LIF biology in aging and warrant systematic investigation.

## 6. Conclusions and Future Perspectives

In summary, skeletal muscle contraction induces the secretion of LIF, a pleiotropic myokine that activates three core downstream signaling cascades. These cascades include JAK2–STAT3, PI3K–Akt1–mTOR, and MEK–ERK1/2. Together, they upregulate the proliferation-associated transcription factors JunB and c-Myc, promote myoblast proliferation and survival, inhibit apoptosis, and ultimately drive physiological skeletal muscle hypertrophy. Myogenic LIF acts primarily via local autocrine/paracrine signaling on skeletal muscle itself, but may also exert systemic endocrine effects on distant organs; preclinical studies have demonstrated that intramuscular injection of LIF-cDNA plasmids elevates circulating LIF levels to promote tissue repair in injured organs. Notably, exercise-induced LIF is rarely detectable in the systemic circulation, likely due to rapid local uptake by skeletal muscle to meet the demands of adaptive remodeling. Preclinical studies have evaluated sustained LIF overexpression in the diaphragm of dystrophic mice. Exogenous LIF administration upregulates dystrophin expression in mdx mice, enhances the survival of transplanted myoblasts, and protects dystrophic myofibers from necrosis via suppression of pro-inflammatory signaling. These findings position LIF/LIFR signaling as a promising therapeutic target for muscular dystrophies, sarcopenia, and other muscle atrophy disorders, providing a theoretical foundation for the development of exercise-based rehabilitation strategies.

Future investigations should focus on identifying optimal exercise modalities and intensities to achieve maximal activation of the skeletal muscle endocrine system, thereby providing new avenues for the treatment of musculoskeletal disorders. Elucidating the endocrine regulatory mechanisms of skeletal muscle and exploring relevant biological targets holds significant theoretical and clinical importance for improving musculoskeletal system function and advancing exercise-based rehabilitation strategies for various systemic diseases. Future investigations should also explore the crosstalk between LIF signaling and free fatty acid metabolism in the regulation of vascular function and skeletal muscle homeostasis, as well as the pharmacokinetics of LIF in aging, including half-life, clearance, and receptor-mediated endocytosis.

## Figures and Tables

**Table 1:** LIF by exercise modality and training volume.

Exercise Modality	Exercise Duration and Intensity	Species	Muscle Type/Sample Site	Results	Reference
Voluntary wheel running	Free wheel running for 2 weeks	Mouse	Tibialis anterior and diaphragm muscles	Post-exercise LIF increase promotes myoblast survival in dystrophic muscle via anti-inflammation	[72]
High-intensity resistance training	Alternating leg press and knee extension exercises	Human	Vastus lateralis muscle	LIF acts as contraction-induced myokine to promote satellite cell proliferation autocrine paracrine	[35]
Electrical pulse stimulation	14 V, 5 Hz, 2 ms	Human	Vastus lateralis myotubes	Skeletal muscle is a contractile activity-regulated endocrine organ expressing myokines, including LIF	[97]
Interval exercise training	Alternating 7 min at 25 m/min (85–90% VO2max) and 3 min at 15 m/min (50–60% VO2max) for 8 weeks	Rat	Gastrocnemius muscle	LIF/LIFR up STAT3 activation reduced cardiac fibrosis, less muscle apoptosis atrophy	[60]
Resistance training	Eight constant-load exercises targeting major muscle groups, performed three times weekly for 14 weeks	Human	Vastus lateralis muscle	Higher post-exercise LIF expression correlates with macrophage MMP14 changes	[72]
Electrical pulse stimulation	12 V, 1 Hz, 2 ms, 24 h	Human	Vastus lateralis myotubes	Myotube LIF upregulation and LIF drive macrophage-mediated ECM remodeling	[97]
Static/dynamic exercise	knee extension held to exhaustion at 50% of deadlift one-rep max; Dynamic: cycle ergometer (5 min exercise, 3 min rest, 5 min exercise)	Human	Plasma	Dynamic exercise had no effect on plasma LIF, whereas static loading increased plasma LIF by 50%	[101]
Treadmill training	Short-duration (10 min/day) or long-duration (55 min/day) treadmill running for 14 sessions (6 days/week, 18 m/min, 5% incline)	Mouse	Plasma	Long-duration exercise increased plasma LIF by 33% relative to sedentary controls, whereas short-duration exercise had no significant effect	[98]

## Data Availability

No new data were created or analyzed in this study.

## Abbreviations

LIF	Leukemia Inhibitory Factor
LIFRβ	LIF Receptor β (gp190)
sLIFR	Soluble LIF Receptor
gp130 (IL6ST)	Glycoprotein 130
sgp130	Soluble gp130
IL-6	Interleukin-6
IL-1β	Interleukin-1β
IL-4	Interleukin-4
IL-8	Interleukin-8
IL-15	Interleukin-15
JAK	Janus Kinase
JAK1/JAK2	Janus Kinase 1/2
TYK2	Tyrosine Kinase 2
STAT1/STAT3/STAT5	Signal Transducer and Activator of Transcription 1/3/5
SOCS3	Suppressor of Cytokine Signaling 3
SHP2	Src Homology-2 Domain-Containing Phosphatase 2
PI3K	Phosphoinositide 3-Kinase
Akt	Protein Kinase B
mTOR/mTORC1	Mechanistic Target of Rapamycin (Complex 1)
MEK	Mitogen-Activated Protein Kinase Kinase
ERK1/2	Extracellular Signal-Regulated Kinase 1/2
MAPK	Mitogen-Activated Protein Kinase
p38 MAPK	p38 Mitogen-Activated Protein Kinase
AMPK	AMP-Activated Protein Kinase
BACH1	BTB and CNC Homology 1
OSM	Oncostatin M
OSMR	Oncostatin M Receptor
CT-1	Cardiotrophin-1
CNTF	Ciliary Neurotrophic Factor
CNTFRα	CNTF Receptor α
CLCF1	Cardiotrophin-Like Cytokine Factor 1
CRLF1	Cytokine Receptor-Like Factor 1
IGF-1	Insulin-Like Growth Factor-1
TNF-α	Tumor Necrosis Factor-α
GDF15	Growth Differentiation Factor 15
MMP14	Matrix Metalloproteinase 14
ECM	Extracellular Matrix
MyoD	Myogenic Differentiation Antigen
JunB	Jun B Proto-Oncogene
c-Myc	Cellular Myelocytomatosis Oncogene
Nr4a3	Nuclear Receptor Subfamily 4 Group A Member 3
LACTB	Lactamase Beta
MIB1	MIB E3 Ubiquitin Protein Ligase 1
UBR4	Ubiquitin Protein Ligase E3 Component N-Recognin 4
VPS13A	Vacuolar Protein Sorting 13 Homolog A
NPL	N-Acetylneuraminate Pyruvate Lyase
sIL-6R	Soluble IL-6 Receptor
miR-29c	MicroRNA-29c
PoWeR	Progressive Weighted Wheel Running
mdx	X-Linked Muscular Dystrophy (mouse model)
MI	Myocardial Infarction
VO2max	Maximum Oxygen Uptake
EPS	Electrical Pulse Stimulation
TA	Tibialis Anterior
VL	Vastus Lateralis
